# Editorial: The current state of knowledge on asthma-COPD overlap (ACO) in clinical and experimental research: what do we know so far?

**DOI:** 10.3389/fimmu.2025.1635488

**Published:** 2025-06-10

**Authors:** Fernanda Degobbi Tenorio Quirino dos Santos Lopes, Iolanda de Fátima Lopes Calvo Tibério, Renato Fraga Righetti

**Affiliations:** ^1^ Laboratory of Experimental Therapeutics, LIM-20, Department of Clinical Medicine, School of Medicine, University of Sao Paulo, Sao Paulo, Brazil; ^2^ Thoracic Surgery Research Laboratory (LIM-61), Division of Thoracic Surgery, Heart Institute (InCor), Clinics Hospital, School of Medicine, University of Sao Paulo, Sao Paulo, Brazil

**Keywords:** ACO, COPD - chronic obstructive pulmonary disease, asthma, molecular pathway mechanism, therapeutic targets

Since Gibson et al. (1) introduced the concept of asthma-COPD overlap (ACO) in 2009, various guidelines have proposed different definitions (2). Most identify key criteria: airflow obstruction that is not completely reversible, asthma diagnosed before age 40, chronic symptoms that fluctuate and worsen, and a history of smoking (≥10 years) or significant exposure to harmful airborne substances (2, 3).

The ACO umbrella encompasses subgroups such as smoking-related COPD with underlying type 2 (T2) inflammation, typically indicated by elevated eosinophils in blood or sputum (2, 4–6). Some individuals with prior asthma diagnoses and significant smoking histories may later develop COPD features, including partially irreversible airflow obstruction (7, 8).

Currently, ACO treatment is extrapolated from asthma and COPD studies. Effective therapies for ACO remain ill-defined due to the lack of consensus on its definition. Additionally, clinical studies often exclude patients with overlapping asthma and COPD features, hindering evidence-based management development.

Further research is needed to develop safer, more effective treatments grounded in a better understanding of ACO. Recent studies have highlighted new molecular pathways in ACO patients and subgroups, reinforcing the roles of smoking and genetics in exacerbating inflammatory conditions.

Sunata et al. analyzed inflammatory properties of eosinophils in ACO and eosinophilic COPD (eCOPD) by isolating cells from healthy individuals, non-eCOPD patients, and ACO/eCOPD patients. Multi-omics analyses (transcriptomics, proteomics, lipidomics) revealed similarities between ACO and eCOPD involving antiviral responses and cholesterol metabolism. Cytokines like IL-33, TNF-α, and IFN-γ activate eosinophils, particularly via viral infections, through mechanisms distinct from the IL-5 pathway. Altered COX metabolism, especially in ACO/eCOPD, and the regulatory role of PGE2 were also noted. *In vitro*, statins, alongside corticosteroids, suppressed inflammatory responses in ACO and eCOPD.

Epithelial to mesenchymal transition (EMT) is a cellular process where epithelial cells lose adhesion and gain migratory mesenchymal characteristics, often due to microenvironmental signals. Dey et al. previously reported structural changes linked to EMT in COPD, but evidence in ACO lungs was lacking. Considering smoking and chronic inflammation in ACO could inflict epithelial injury, they investigated EMT in ACO patients using large airway endobronchial biopsy (EBB) tissues from asthma, COPD current smokers (CS), ex-smokers (ES), and ACO patients, compared to healthy controls (HC) and current smokers with normal lung function (NLFS). They observed in ACO patients significant RBM fragmentation, reduced E-cadherin, increased N-cadherin expression, and elevated vimentin and S100A4-positive basal cells, confirming EMT occurrence. Most parameters showed similar changes between COPD-CS and ACO patients.

Previous studies also linked extracellular matrix (ECM) remodeling with ACO. Camargo et al. (9) demonstrated that IL-17 pathway inhibition significantly reduced ECM remodeling and airway obstruction in an experimental ACO model, underscoring ECM’s pivotal role in disease progression.

Gutiérrez-Romero et al. highlighted genetic factors that increase Th2-like chemokine levels in COPD, a disease typically associated with Th17 and Th1 responses. Also, they reinforced the importance of air pollutants in this disease progression. They demonstrated in a Mexican population that a TLR4 gene single-nucleotide polymorphism (SNP) amplified chronic inflammation—marked by IFN-γ, IL-4, and IL-5 in COPD smokers—compared to COPD secondary to biomass-burning smoke, which showed elevated IL-6, IFN-γ, and IL-10, mediating distinct inflammatory responses.

Emerging evidence also implicates localized autoimmune dysfunction within airways in asthma development (10). Qin et al. demonstrated significant associations between airway eosinophilic inflammation, sputum immunoglobulin G (Sp-IgG), and asthma severity. Sp-IgG was found to induce eosinophil cytolytic extracellular trap cell death, suggesting a potential autoimmune mechanism in asthma. Using clinical and Mendelian randomization analyses, they underscored eosinophil-mediated IgG’s role in asthma severity and identified a panel of Sp-IgG epitopes (Sp-IgGEPs) as potential biomarkers reflecting airway autoimmune events in asthma.

In summary, these findings emphasize the importance of innovative multi-omics approaches and translational models in elucidating distinct immunological and metabolic signatures among ACO subgroups. This points to the necessity of more personalized therapeutic interventions. Discoveries such as eosinophil activation via non-IL-5 pathways, the involvement of antiviral responses and cholesterol metabolism, and epithelial injury identified through EMT markers highlight the multifactorial nature of ACO.

Coordinated and inclusive clinical research is essential for advancing ACO understanding. Generating robust evidence to support precision medicine, through molecular biomarker identification and individualized therapeutic strategies, will be key to overcoming current limitations and improving outcomes for patients affected by this complex, often underrecognized condition.

## References

[1] Gibson PG Simpson JL. The overlap syndrome of asthma and COPD: what are its features and how important is it? Thorax. (2009) 64:728–35. doi: 10.1136/thx.2008.108027 19638566

[2] Fouka E Papaioannou AI Hillas G Steiropoulos P. Asthma-COPD overlap syndrome: recent insights and unanswered questions. J Pers Med. (2022) 12:708. doi: 10.3390/jpm12050708 35629128 PMC9146831

[3] Leung C Sin DD. Asthma-COPD overlap: what are the important questions? Chest. (2022) 161:330–44. doi: 10.1016/j.chest.2021.09.036 34626594

[4] Christenson SA Steiling K van den Berge M Hijazi K Hiemstra PS Postma DS. Asthma-COPD overlap. Clinical relevance of genomic signatures of type 2 inflammation in chronic obstructive pulmonary disease. Am J Respir Crit Care Med. (2015) 191:758–66. doi: 10.1164/rccm.201408-1458OC PMC4407484 25611785

[5] Bonten TN Kasteleyn MJ de Mutsert R Hiemstra PS Rosendaal FR Chavannes NH. Defining asthma-COPD overlap syndrome: a population-based study. Eur Respir J. (2017) 49:1602008. doi: 10.1183/13993003.02008-2016 28461292

[6] Barrecheguren M Pinto L Mostafavi-Pour-Manshadi SM Tan WC Li PZ Aaron SD. CanCOLD Collaborative Research Group and the Canadian Respiratory Research Network. Identification and definition of asthma-COPD overlap: The CanCOLD study. Respirology. (2020) 25:836–49. doi: 10.1111/resp.13780 32064708

[7] Boulet LP Boulay MÈ Dérival JL Milot J Lepage J Bilodeau L. Asthma-COPD Overlap Phenotypes and Smoking: Comparative features of asthma in smoking or non-smoking patients with an incomplete reversibility of airway obstruction. COPD. (2018) 15:130–8. doi: 10.1080/15412555.2017.1395834 29683758

[8] Soler-Cataluña JJ Novella L Soler C Nieto ML Esteban V Sánchez-Toril F. Clinical characteristics and risk of exacerbations associated with different diagnostic criteria of asthma-COPD overlap. Arch Bronconeumol (Engl Ed). (2020) 56:282–90. doi: 10.1016/j.arbres.2019.08.023 31784349

[9] Camargo LDN Righetti RF de Almeida FM Dos Santos TM Fukuzaki S Martins NAB. Modulating asthma-COPD overlap responses with IL-17 inhibition. Front Immunol. (2023) 14:1271342. doi: 10.3389/fimmu.2023.1271342 37965351 PMC10641519

[10] Salter B Zhao N Son K Tan NS Dvorkin-Gheva A Radford K. Airway autoantibodies are determinants of asthma severity. Eur Respir J. (2022) 60:2200442. doi: 10.1183/13993003.00442-2022 35777765

